# Biological Behavior of Papillary Carcinoma of the Thyroid Including Squamous Cell Carcinoma Components and Prognosis of Patients Who Underwent Locally Curative Surgery

**DOI:** 10.1155/2012/230283

**Published:** 2011-09-18

**Authors:** Yasuhiro Ito, Mitsuyoshi Hirokawa, Takuya Higashiyama, Minoru Kihara, Chisato Tomoda, Yuuki Takamura, Kaoru Kobayashi, Akihiro Miya, Akira Miyauchi

**Affiliations:** ^1^Department of Surgery, Kuma Hospital, Shimoyamate-Dori, Chuo-Ku, Kobe 650-0011, Japan; ^2^Department of Pathology, Kuma Hospital, Kobe 650-0011, Japan

## Abstract

Thyroid carcinoma showing squamous differentiation throughout the entire lesion is diagnosed as squamous cell carcinoma of the thyroid (SCCT) in the WHO classification. This entity is a rare disease and shows a dire prognosis; however, squamous differentiation is more frequently detected in only a portion of papillary thyroid carcinoma. In this paper, we present our experience of 10 patients (8 primary lesions and 2 with recurrence in the lymph nodes) with papillary thyroid carcinoma having an SCC component (PTC-SCC). Only 3 of 8 primary lesions (38%) and none of the 2 recurrent nodes were preoperatively diagnosed as or suspected of having SCC components. All 10 patients underwent locally curative surgery. To date, 3 patients have died of carcinoma, and 2 had distant metastasis at diagnosis or had an undifferentiated carcinoma component. The other 7 are currently alive 5 to 43 months after diagnosis. Systemic adjuvant therapy after the detection of recurrence was effective for 2 patients. It is possible that some PTC-SCC patients without distant metastasis who undergo locally curative surgery can survive for a prolonged period and adjuvant therapies can be effective for local and distant recurrences.

## 1. Introduction

Squamous cell carcinoma of the thyroid (SCCT) is known to show very aggressive characteristics and a dire prognosis. In the WHO classification [1], SCCT is defined as “squamous cell carcinoma of the thyroid should be composed entirely of tumour cells with squamous differentiation.” According to this definition, SCCT is an extremely rare disease [2–5]. However, squamous differentiation can be more frequently observed as a SCC component in a portion of the papillary carcinoma (PTC) lesion, especially in the tall cell variant [6]. Most previous studies investigating a comparably large number of patients investigated SCCT and PTC with an SCC component (PTC-SCC) as a single group, and our knowledge regarding the biological characteristics of PTC-SCC remains poor because of its rarity. To date, we have encountered 10 patients who were diagnosed as having PTC-SCC on postoperative pathological examination. In this study, we present their clinicopathological features, therapies, and clinical outcomes to elucidate the biological behavior of this disease.

## 2. Patients and Methods

We reviewed the surgical and pathological records of patients who underwent surgery for thyroid carcinoma between 2006 and 2010. In this period, 5749 patients underwent surgery for primary or recurred PTC. Of these, 10 patients (0.2%) were diagnosed as PTC-SCC and were enrolled in this study. In this period, no patients were diagnosed as SCCT. These diagnoses were rereviewed by a thyroid pathologist (M.H.) based on the WHO classification [1]. PTC-SCC was diagnosed in 8 primary lesions obtained at initial surgery and 2 recurrent lymph nodes obtained at second surgery. Patients who had SCC sites other than the thyroid were excluded from the series. Patients who were diagnosed as having PTC with squamous cell metaplasia (squamous lesions appear benign) or squamoid subtype in undifferentiated carcinoma (UC) were excluded from the study. In this period, 3 patients were suspected as having PTC-SCC (1 primary lesion and 2 recurring lesions) but underwent biopsy only. One patient died of carcinoma one month after detection, and two patients were referred to other hospitals, and their prognoses are unknown. These patients were also excluded from the study. In our department, all patients with PTC undergo thyroid ultrasonography, and neck CT scan is also performed for those who have clinical lymph node metastasis and whose primary lesions are suspected of having extension to adjacent organs. All patients in our series also underwent ultrasonography. Neck CT scan (plain and enhanced) was performed for 9 patients. Furthermore, all patients underwent chest CT scan to screen for lung metastasis and 3 patients who were cytologically suspected of having SCC or UC also underwent abdominal CT scan also. None of these patients underwent PET-CT preoperatively, although two underwent PET-CT after surgery. All patients underwent ultrasound-guided fine needle aspiration biopsy (FNAB) for diagnosis. Furthermore, 3 patients who were suspected of SCC or UC on cytology underwent also ultrasound-guided core needle biopsy (CNB) to confirm the diagnosis. None of these patients showed any complications of CNB, such as hemorrhage, hematoma, and recurrent laryngeal nerve paralysis. On pathological examination, UC lesion was also detected in 2 patients (one primary lesion and one recurrent lymph node). One patient also had primary lung carcinoma, which was detected on CT scan before thyroid surgery. After thyroid surgery, this patient was referred to another hospital and underwent surgery and chemotherapy for lung carcinoma. The histology of lung carcinoma was adenocarcinoma.

## 3. Results

We analyzed the backgrounds and preoperative findings on imaging studies of 10 PTC-SCC (Table 1). Patients consisted of 7 females and 3 males, and patient ages ranged from 68 to 83 years. Of 8 patients who underwent initial surgery, only 3 (38%) (patient nos. 1, 4, and 8) were preoperatively diagnosed as or suspected of having an SCC component on FNAB or CNB. Tumors of these 3 patients were suspected to significantly extend to adjacent organs. Therefore, these patients underwent chemotherapy using paclitaxel [7] before surgery, considering the possibility of UC with Stage IVB [8], to promote tumor shrinkage and make locally curative surgery easier. There was no evidence of carcinoma progression during preoperative chemotherapy, *although no patients were judged as complete response* (manuscript in preparation). The remaining 5 primary lesions were diagnosed as PTC on cytology. Tumor size ranged from 3.5 to 5.6 cm, and 7 of these patients also had lymph node metastasis detected on preoperative imaging studies. Three patients (patient nos. 6, 7, and 8) also underwent FNAB for metastatic nodes and were diagnosed as or suspected of having PTC. Two patients (patients nos. 2 and 3) had previously undergone initial surgery for PTC, and recurrence was detected in the lymph nodes, which measured 0.9 cm and 3.9 cm, respectively. The cytological results of these nodes were PTC and UC, respectively. Two patients (patient nos. 1 and 3) had lung metastasis at surgery, and one (patient no. 10) had primary lung carcinoma that was treated after surgery for thyroid carcinoma at another hospital, as indicated above. 

Table 2 summarizes the intraoperative and postoperative findings and clinical courses of these 10 patients. All 8 primary lesions showed significant extrathyroid extension corresponding to T4a in the UICC TNM classification [8], but all these patients could undergo locally curative surgery. Of 8 patients, 2 (patient nos. 4 and 5) were SCC dominant, and the remaining 6 (patient nos. 1, 6–10) were PTC dominant on pathological examination for primary lesions. PTC lesion was dominant in 2 patients who underwent surgery for lymph node recurrence (patient nos. 2 and 3). The range of the portion of SCC component was less than 10% in 2 patients (patient nos. 2 and 6), 10–20% in 5 patients (patient nos. 1, 7–10), and 40% or more in 3 patients (patient nos. 3–5). The PTC lesions of 4 patients (50%) were tall cell variant. Two patients (patient nos. 3 and 4) also demonstrated UC lesion. 

 None of these 10 patients underwent radioactive iodine (RAI) therapy after surgery. The 3 patients who underwent preoperative chemotherapy continued to receive it after surgery. Chemotherapy was initiated after surgery for patient nos. 3 and 5. Patient no. 3 underwent chemotherapy immediately after initial surgery because of lung metastasis and involvement of a UC component and patient no. 5 underwent chemotherapy after the detection of local recurrence. Patient no. 9 showed recurrence in the lung 3 months after surgery and underwent immunotherapy using dendritic cells pulsed with WT1 peptide at the patient's request [9]. Three patients (patients nos. 5, 7, and 8) underwent external beam radiotherapy (EBRT) of the whole neck at 50–60 Gy immediately after surgery. However, 2 local recurrences were detected during EBRT in patient no. 5. One was located in front of the common carotid artery and was diagnosed as SCC on CNB, and another was found in the skin, for which neither FNAB nor CNB was performed. The former disappeared by postoperative chemotherapy, and the latter was resected by second surgery. The histology of the second surgical specimen was SCC.

 During followup, patient no. 1, who had lung metastasis at surgery, developed recurrence in the bone and died of carcinoma 18 months after diagnosis because of the enlargement of lung metastasis. Patient no. 3 is currently alive despite the development of lung metastasis that had been present before surgery. Patient no. 4, who had a UC component, developed recurrence in the larynx and underwent a second surgery at another hospital. The recurrent tumor was pathologically diagnosed as UC, and this patient died of UC progression 23 months after diagnosis. In patient no. 5, there has been no further evidence of carcinoma recurrence to date after chemotherapy and second surgery. Lung metastasis was stable in patient no. 9 for 6 months after the initiation of immunotherapy, but he died of sudden respiratory tract hemorrhage 9 months after surgery. The remaining 5 (patients nos. 2, 6–8, 10) are currently alive with no evidence of carcinoma recurrence, although patient no. 10 is currently undergoing chemotherapy for lung carcinoma.

## 4. Discussion

In this study, we demonstrated that it is difficult to diagnose PTC-SCC on preoperative FNAB or CNB. Only 3 of 8 patients (38%) having primary lesions were preoperatively diagnosed as or suspected of having SCC components. This is partially because of the difficulty in differential diagnosis between SCC and UC on cytology. Furthermore, SCC cells may not be aspirated or biopsied if the needle does not appropriately hit SCC components. In our series, all patients were older, 7 patients had carcinoma larger than 4 cm, clinical node metastasis was detected in 7 patients, all had significant extrathyroid extension, and the PTC lesions of 4 patients were diagnosed as tall cell variant. These clinicopathological features were recognized as predicting poor prognosis, indicating that squamous differentiation occurs in PTC with biologically aggressive behavior [10–13]. 

 Previous studies investigated SCCT and PTC-SCC as a single group and demonstrated that most of these patients showed a poor prognosis. Booya et al. showed that the median survival period was only 8.6 months for 10 patients [2]. According to a review by Syed et al. in 2010 [14], median survival periods in reports published between 1985 and 2009 ranged from 3 to 24 months but mostly 12 months or even shorter. Furthermore, Cook et al. demonstrated that all patients with SCCT or PTC-SCC who underwent incomplete resection or biopsy died within a short period despite adjuvant therapies [3], which was similar to the results of UC, as we previously demonstrated [15]. 

 In this study, we investigated 10 patients with PTC-SCC who underwent locally curative resection, indicating that they were potential long-term survivors. In our series, 7 patients have survived for 8 months to 43 months to date. Two patients (patients nos. 2 and 6) with only focal SCC lesions who did not show distant metastasis at diagnosis have survived without any further recurrence for 14 and 43 months after diagnosis, respectively, although they underwent no adjuvant therapies. The remaining 5 with larger lesions of SCC components, who underwent either or both chemotherapy and EBRT, have also survived with or without recurrence. Of the 3 patients who died of carcinoma, 2 had distant metastasis at diagnosis or had a UC component. These findings suggest that long-term survival can be expected for some PTC-SCC patients if they undergo locally curative surgery and do not have distant metastasis at diagnosis or a UC component. 

 Previous studies demonstrated controversial results of adjuvant chemotherapy [16–18]. In our series, 3 patients who were cytologically diagnosed as or suspected of having SCC underwent preoperative adjuvant chemotherapy. These tumors were large and were suspected of extending to adjacent organs. In order to make locally curative surgery easier, we performed preoperative chemotherapy based on the protocol for UC [7]. If a tumor is small without the suspicion of extrathyroid extension and locally curative surgery is expected to be easy, immediate surgery may be preferred to avoid losing time. Postoperative chemotherapy should be performed for patients having distant metastasis at surgery or those who have undergone only palliative surgery. It remains unclear whether chemotherapy immediately after locally curative surgery for patients without distant metastasis is effective to prolong the survival of PTC-SCC patients, because we did not perform a comparative study. However, it may be an alternative in order to control minute carcinoma lesions that remained unresected in local lesions and micrometastases in distant organs. Another strategy is to initiate chemotherapy when recurrence is clinically detected. Since one of the local recurrences disappeared in patient no. 5, this may also promise a certain level of effect.

 EBRT is another adjuvant therapy for local control. However, previous studies of SCCT and PTC-SCC showed controversial findings of its effectiveness [2, 3, 18, 19]. In our series, 3 patients underwent EBRT immediately after surgery. Two patients showed no evidence of local recurrence, but in 1 patient, local recurrences became apparent during EBRT. Therefore, we cannot conclude that EBRT has a significant effect on the local control of PTC-SCC. However, it may be better to perform postoperative EBRT, especially for cases with significant extension to adjacent organs, because multimodality therapy should be considered for aggressive diseases such as PTC-SCC. 

 In summary, we have presented our experience of treating 10 patients with PTC-SCC, which is difficult to diagnose preoperatively. Long-term survival can be expected for PTC-SCC patients if locally curative resection can be performed and distant metastasis at surgery is not detected. Adjuvant chemotherapy and EBRT may contribute to the control of carcinoma recurrence, but further studies of a large number of patients are necessary to establish therapeutic protocols for PTC-SCC patients.

## Figures and Tables

**Table 1:** Backgrounds and preoperative evaluation of 10 patients with PTC-SCC.

Patient no.	Gender	Age	Initial surgery?	Rapid growth	*Preoperative diagnosis (primary)	*Preoperative diagnosis (LN meta)	Tumor size (cm)	LN meta size (cm)	Distant metastasis at surgery
1	Female	71	Yes	Yes?	SCC	x	5.6	1.6	Lung
2	Female	71	No	No	x	PTC	x	0.9	No
3	Female	69	No	Yes	x	UC	x	3.9	Lung
4	Female	67	Yes	No	UC or SCC	x	4.9	x	No
5	Female	83	Yes	No	PTC or PDC	x	5.2	1.7	No
6	Male	76	Yes	No	PTC	PTC	4.0	1.4	No
7	Male	71	Yes	No	PTC	PTC	3.5	0.9	No
8	Female	68	Yes	No	PTC or SCC	PTC?	4.4	2.7	No
9	Male	70	Yes	No	PTC	x	4.4	3.6	No
^∧^10	Female	71	Yes	No	PTC	x	4.7	2.7	No

PTC: papillary thyroid carcinoma, UC: undifferentiated carcinoma; PDC: poorly differentiated carcinoma, SCC: squamous cell carcinoma.

*Based on FNAB or CNB.

^∧^Also had lung carcinoma.

**Table 2:** Intraoperative and pathological findings and prognosis of 10 patients with PTC-SCC.

Patient no.	Surgical designs	Resection	Extrathyroid extension	Pathology (primary)	Pathology (LN meta)	Adjuvant therapies	Carcinoma recurrence	Outcome after diagnosis
1	TT + MND	R0?	Yes	**PTC > SCC (10%)	PTC	*chemo	Bone	18 m DOC
2	CT + MND	R0	x	x	PTC > SCC (<10%)	x	x	43 m ANEC
3	MND	R0	x	x	PTC > SCC (40%) > UC	Chemo	x	33 m AWC
4	TT + CND	R0	Yes	SCC (40%) > UC > PTC	PTC	*chemo	^∧^Laryn, Bone	23 m DOC
5	TT + MND	R0	Yes	SCC (80%) > PTC (tall)	PTC	Chemo + EBRT	Skin, local	18 m ANEC
6	TT + MND	R0?	Yes	PTC > SCC (<10%)	PTC	x	x	14 m ANEC
7	TT + MND	R0	Yes	PTC (tall) > SCC (40%)	PTC	EBRT	x	13 m ANEC
8	LI + MND	R0	Yes	PTC (tall) > SCC (10%)	PTC	*chemo + EBRT	x	9 m ANEC
9	TT + MND	R0	Yes	PTC (tall) > SCC (20%)	PTC	Immunotherapy	Lung	9 m DOC
10	TT + MMD	R1	Yes	PTC > SCC (20%)	PTC	Chemo for lung carcinoma	x	5 m ANEC

*Preoperative chemotherapy was also performed. ^∧^Recurrence of UC. **A > B indicates A occupied a larger portion than B.

TT: total thyroidectomy, CT: completion total thyroidectomy, LI: lobectomy with is thymectomy, CND: central node dissection, MND: modified radical neck dissection, EBRT; external beam radiotherapy.

DOC; Died of carcinoma.

ANEC: Alive with no evidence of Carcinoma.

AWC: Alive with carcinoma.
